# High-throughput analysis of the *Trypanosoma cruzi* minicirculome (mcDNA) unveils structural variation and functional diversity

**DOI:** 10.1038/s41598-024-56076-4

**Published:** 2024-03-07

**Authors:** Andrés Gómez-Palacio, Lissa Cruz-Saavedra, Frederik Van den Broeck, Manon Geerts, Sebastián Pita, Gustavo A. Vallejo, Julio C. Carranza, Juan David Ramírez

**Affiliations:** 1 Laboratorio de Investigación en Genética Evolutiva, Universidad Pedagógica y Tecnológica de Colombia, Boyacá, Colombia; 2 Centro de Investigaciones en Microbiología y Biotecnología-UR (CIMBIUR), Facultad de Ciencias Naturales, Universidad del Rosario, Bogotá, Colombia; 3 Department of Microbiology, Immunology and Transplantation, Rega Institute for Medical Research, KU Leuven, 3000 Leuven, Belgium; 4 Department of Biomedical Sciences, Institute of Tropical Medicine, 2000 Antwerp, Belgium; 5 Fish Eco-Evo-Devo and Conservation, KU Leuven, 3000 Leuven, Belgium; 6 Directorate Taxonomy and Phylogeny, Royal Belgian Institute for Natural Sciences, 1000 Brussels, Belgium; 7 Sección Genética Evolutiva, Facultad de Ciencias, Universidad de La República, Montevideo, Uruguay; 8 Laboratorio de Investigación en Parasitología Tropical, Facultad de Ciencias, Universidad del Tolima, Ibagué, Colombia; 9 Molecular Microbiology Laboratory, Department of Pathology, Molecular and Cell-Based Medicine, Icahn School of Medicine at Mount Sinai, New York City, NY 10029 USA

**Keywords:** *Trypanosoma cruzi*, Maxicircles, Minicircles, kDNA, Guide RNA, Colombia, Chagas disease, Microbial genetics, Parasitology

## Abstract

*Trypanosoma cruzi* causes Chagas disease and has a unique extranuclear genome enclosed in a structure called the kinetoplast, which contains circular genomes known as maxi- and minicircles. While the structure and function of maxicircles are well-understood, many aspects of minicircles remain to be discovered. Here, we performed a high-throughput analysis of the minicirculome (mcDNA) in 50 clones isolated from Colombia’s diverse *T. cruzi* I populations. Results indicate that mcDNA comprises four diverse subpopulations with different structures, lengths, and numbers of interspersed semi-conserved (previously termed ultra-conserved regions mHCV) and hypervariable (mHVPs) regions. Analysis of mcDNA ancestry and inter-clone differentiation indicates the interbreeding of minicircle sequence classes is placed along diverse strains and hosts. These results support evidence of the multiclonal dynamics and random bi-parental segregation. Finally, we disclosed the guide RNA repertoire encoded by mcDNA at a clonal scale, and several attributes of its abundance and function are discussed.

## Introduction

Mitochondrial DNA (mtDNA) in kinetoplastids is truly unique. It forms a large, intricate structure comprising a complex circular-based genome. This genome consists of numerous maxicircles, typically ranging from 20 to 50 kilobases, and thousands of minicircles, each about 1.4 kilobases in size. These components are intricately woven together in a vast network referred to as the kinetoplast (kDNA)^1^. The advent of high-throughput sequencing has opened up a floodgate of research on kDNA, shedding light on its function, inheritance, diversity, and evolution in various medically important trypanosomes. These include *Leishmania* species^2,3^, *T. brucei* sp.^4–6^, *T. vivax*^7^*, T. lewisi*^8^ and *T. cruzi*^9,10^, among others. While most studies concur that maxicircles are valuable for identifying species or strains and for conducting phylogenetic studies, there is emerging evidence of heteroplasmy and genome-introgression in *T. cruzi*. This suggests that genetic recombination, such as clonal hybridization, has been previously underestimated in natural populations. Consequently, there remains a pressing need for further research into kDNA, including minicircles^11,12^.

Further research into kDNA, including minicircles, is imperative to advance our understanding of *Trypanosoma cruzi*, the causative agent of Chagas disease. This parasite has been classified into seven genetic lineages known as Discrete Typing Units (DTUs), ranging from TcI to TcVI plus TcBat^13,14^. The minicircles present in *T. cruzi*'s minicirculome (mcDNA) add an additional layer of complexity as they play a critical role in post-transcriptional editing of maxicircle mRNA through a process called RNA editing. This process involves the insertion and deletion of uridine in mRNA, for which a tailored repertoire of guide RNAs (gRNAs) is essential. These gRNAs are encoded in AT-rich hypervariable regions (mHVRs) of the minicircles^15–17^. To comprehensively understand the abundance, structure, diversity, inheritance, and evolution of *Trypanosoma* species' minicirculome, it is crucial to conduct studies. The canonical minicircle structure is well-known, consisting of four highly conserved regions (mHCRs) separated by approximately 330 base pairs of mHVRs^18^. However, emerging evidence suggests that a single strain (Y strain) can harbor at least 286 different minicircle sequences, revealing greater heterogeneity than previously thought. These minicircles in TcII (Y strain) and TcV (Bug2148 strain) exhibit distinct sizes and numbers of mHCRs and mHVRs^10^.

Diversity in *T. cruzi*’s mcDNA has been primarily investigated in TcVI (CL Brener) and TcIII (Esmeraldo) strains, uncovering 110 encoded gRNAs and evidence of intra-minicircle recombination compared to publicly available minicircle sequences in GenBank^17^. Recent reports using deep sequencing of mHVR amplicons have shown variable clusters of mHVRs across strains, ranging from 71 in TcV (Mncl2 strain) to 373 in TcIII (X109/2 strain). Some of these clusters are shared among strains both intra and within lineages, hinting at potential bi-parental inheritance of minicircles^12,19^. This diversity in gRNAs repertoire may contribute to the extreme diversity observed in different lineages of *T. cruzi*^15^.

Genotyping of strains can be misleading due to multiclonality, emphasizing the importance of genetic studies on clones to obtain more accurate information about diversity, genome composition, and evolutionary traits in natural *T. cruzi* populations^20,21^. Recent research on the nuclear genome structure and chromosome plasticity in TcI, conducted in 33 clones from diverse host-infecting populations in Colombia and other South American countries, revealed striking evidence of segmental aneuploidy (SA) along chromosomes and loss of heterozygosity (LOH) events even between clones from the same strain^22^. Given the wealth of information obtainable from kDNA and the ongoing debate about mcDNA structure in *T. cruzi*, a comprehensive study was conducted on 50 *T. cruzi* TcI clones. This research has expanded our knowledge about minicirculome evolution, encompassing inheritance, diversity, and intra-genome dynamics, shedding new light on patterns involved in the parasite diversity of Colombian populations^22^.

In this study, we examined minicircles and maxicircles in *T. cruzi* TcI clones from different hosts and strains. We found low phylogenetic resolution in the maxicircle-based tree, with two distinct clades. Minicircle analysis revealed varying numbers per clone, with no significant host-related differences. Minicircle abundance ranged from one to over 50 copies, and sizes varied from 331 to 1415 bp, grouped into four categories. Nucleotide composition and conserved regions differed across minicircle groups, indicating diversity. We identified unique minicircle sets in various clone clusters. Additionally, we predicted gRNAs for gene editing in minicircles, showing variations among clones from different strains and hosts. This study uncovers the complexity of minicircles in TcI clones of *T. cruzi*.

## Results

### The maxicircle-based tree shows low phylogenetic resolution among several TcI clones from distinct hosts

**Figure 1:**
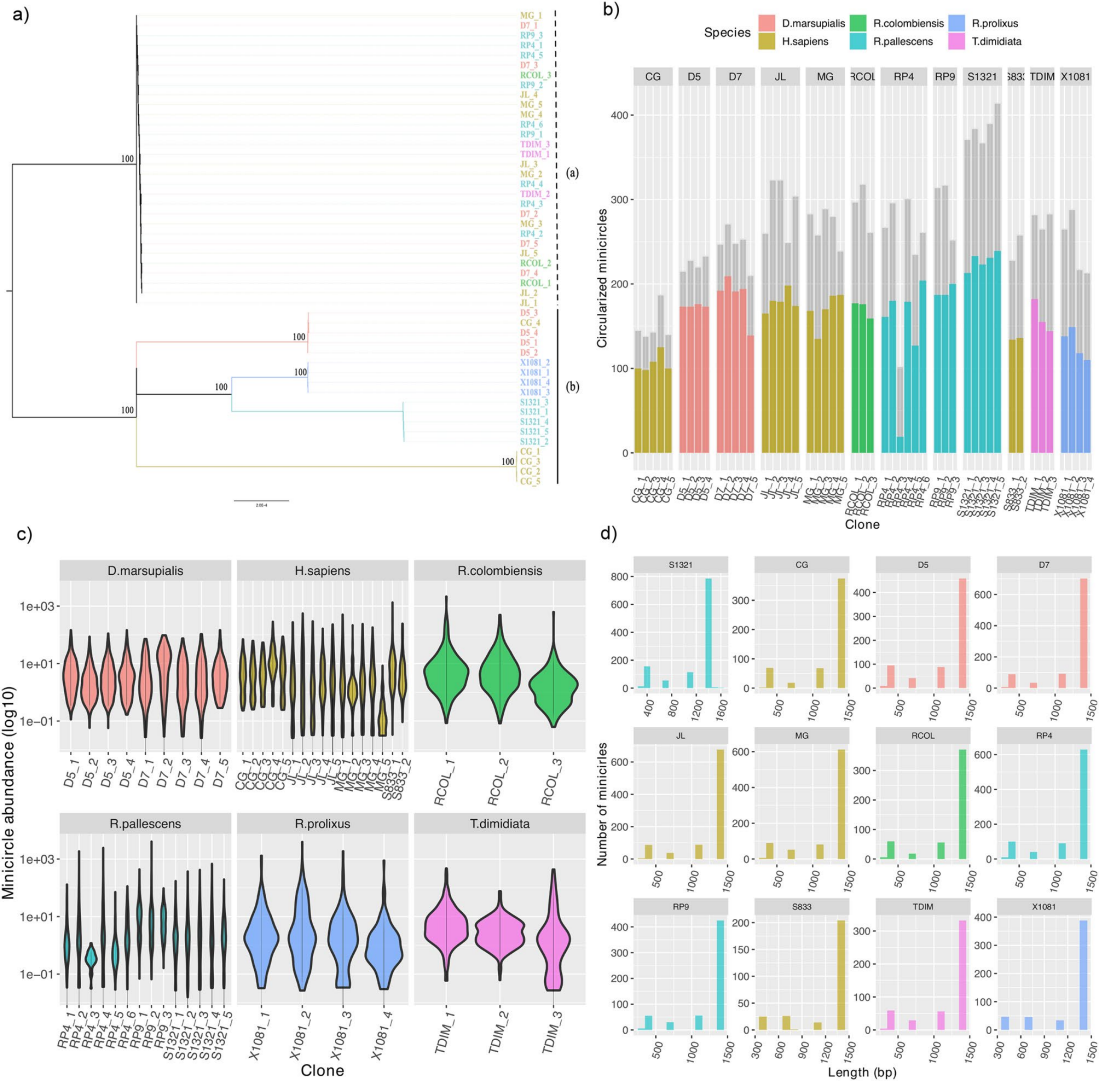
Maxicircle-based tree and the number of minicircles identified in this study. (**a**) Maximum likelihood tree based on maxicircles assembled of TcI clones from Colombia isolated from different hosts as detailed in the inset. Colored branches indicate a monophyletic clade from the same strain (except for CG_4). Bootstrap support is indicated above the branches of the tree. (**b**) Number of assembled (grey) and circularized minicircles obtained in 50 TcI clones. (**c**) Minicircle abundance per cell (i.e., related to the haploid genome) in TcI clones from distinct host. (**d**) Length distribution (i.e., in base pair) of circularized minicircles length in TcI strains. Colors indicate the *T. cruzi* isolated from distinct human, opossum, and vector species as shown in the inset (Table 1).

In the maxicircle ML tree, depicted in Fig. 1a, two highly supported clades, both with a robust bootstrap value of 100, emerged as significant findings. The first clade, (termed clade (i)), contained a majority of clones originating from a wide range of hosts, including humans, opossums, and various vector species. In contrast, the second clade, (termed clade (ii)), exhibited a more intricate structure, consisting of four well-supported sub-clades. These sub-clades contained clones from specific sources, such as *R. prolixus* (strain X1081),* D. marsupialis* (S1321 and D5), along with one human clone (CG_4), and four out of the five clones from the CG strain, which were originally isolated from humans. This organization within clade (ii) sheds light on the shared evolutionary relationships among these *Trypanosoma cruzi *clones, highlighting distinct patterns of divergence and relatedness based on their maxicircle sequences.

**Table 1:** Host, origin, and number of clones analyzed in this work.

Host	Origin (locality, departament)	Strain	Date of isolation	Number of clones
*Homo sapiens*	Cartagena del Chaira, Caquetá	CG	2003	5*
	Arauca, Arauca	JL	1992	5
	Saravena, Arauca	MG	2004	5
	Pará, Belém**	S833	n.d	2
*Didelphis marsupialis*	Coyaima, Tolima	D5	1998	4*
	Coyaima, Tolima	D7	1998	5
*Rhodnius colombiensis*	Coyaima, Tolima	RCOL	2002	3
*Rhodnius pallescens*	San Sebastián, Magdalena	S1321	2010	5*
	San Sebastián, Magdalena	RP4	2010	6
	San Sebastián, Magdalena	RP9	2010	3
*Rhodnius prolixus*	Maní, Casanare	X1081	1998	4
*Triatoma dimidiata*	Boavita, Boyacá	TDIM	1998	3 (1)*

*Indicates the number of clones previously analyzed (23). **Strain from Brazil.

### Minicirculome characterization in TcI clones

In our analysis of 50 TcI clones, we successfully retrieved a total of 13,132 minicircle contigs, with 8254 of them, accounting for 62.9%, being circularized. On average, each clone had around 263 minicircle contigs, with some variation observed, ranging from 197 in clone RP4_3 to 328 in clone S1321_3, both isolated from *R. pallescens* (Fig. 1b). When considering only the circularized minicircles, the average number per clone was 165, with the range spanning from 19 in RP4_3 to 206 in S1321_3. It is noteworthy that most clones exhibited relatively consistent numbers of circularized minicircles, except for an anomaly observed in clone RP4_3, which had notably fewer circular minicircles. Importantly, we did not find any significant differences when comparing clones isolated from different hosts, including humans, opossums, and vectors (as determined by Kruskal–Wallis’ test, *P* value = 0.06, H statistics = 5.6).

For the entire dataset, we observed that, on average, 4.9% of all mapped reads aligned with a mapping quality greater than 20, with around 14.6% aligning in proper pairs. While there were noticeable variations in the number of mapped reads across all clones (Supplementary Fig. S1), we did not identify a significant correlation between the number of circularized minicircles and the quantity of highly-quality mapped reads per clone (as determined by Pearson’s correlation coefficient, r = 0.165; *P* value = 0.25).

### Minicirculome abundance and length in TcI clones

The average minicircle abundance per cell was calculated at 9.6 copies, determined as the ratio of minicircle depth mapping to the mean depth of haploid gDNA. Our analysis revealed that 45.6% of the minicircles exist as single copies, while the remaining minicircles range in abundance from two to 50 copies (Fig. 1c). A minority of minicircles, comprising only 3.1%, exhibited more than 50 copies within a single clone (Fig. 1d). Although minicircle abundance displayed overall uniformity across the clones, a few clones exhibited notably high minicircle abundance, exceeding 1500 copies, including one Brazilian clone (S833_1) and clones isolated from vector species such as *R. colombiensis*, *R. pallescens*, and *R. prolixus* (Fig. 1c).

Regarding the 8254 circularized minicircles, their sizes ranged from 331 to 1415 bp, in line with expectations for *T. cruzi*. Notably, a small subset of minicircles (25 in total) were found to be double the usual length (~ 2800 bp) and were consequently excluded from subsequent analyses as they were likely artifacts (Supplementary Fig. S2). The distribution of minicircle lengths remained consistent among clones obtained from various strains and hosts (Fig. 1d).

Based on the length of minicircles within the samples, they were categorized into four distinct groups. Group 1 included 998 minicircles with lengths between 331 and 393 bp (average 357 bp), Group 2 consisted of 429 minicircles with lengths between 697 and 751 bp (average 751 bp), Group 3 comprised 837 minicircles ranging from 1059 to 1098 bp in length (average 1073 bp), and Group 4 encompassed the largest number of minicircles, totaling 5965, with lengths between 1409 and 1556 bp (average 1428 bp) (Fig. 1d and Supplementary Fig. S2). Importantly, all four groups were present in samples from all hosts, and the distribution of minicircles was consistent across different strains (Table 2).

**Table 2:** The number of minicircles for groups 1–4 identified in *T. cruzi* I from different hosts.

Host	Group1	Group2	Group3	Group4
	(331–393 bp)	(697–751 bp)	(1059–1098 bp)	(1409–1556 bp)
*H. sapiens*	282	135	251	1866
*D. marsupialis*	200	76	180	1160
Vector	516	218	406	2939
Total	998	429	837	5965

### Minicirculome structure in TcI clones

**Figure 2:**
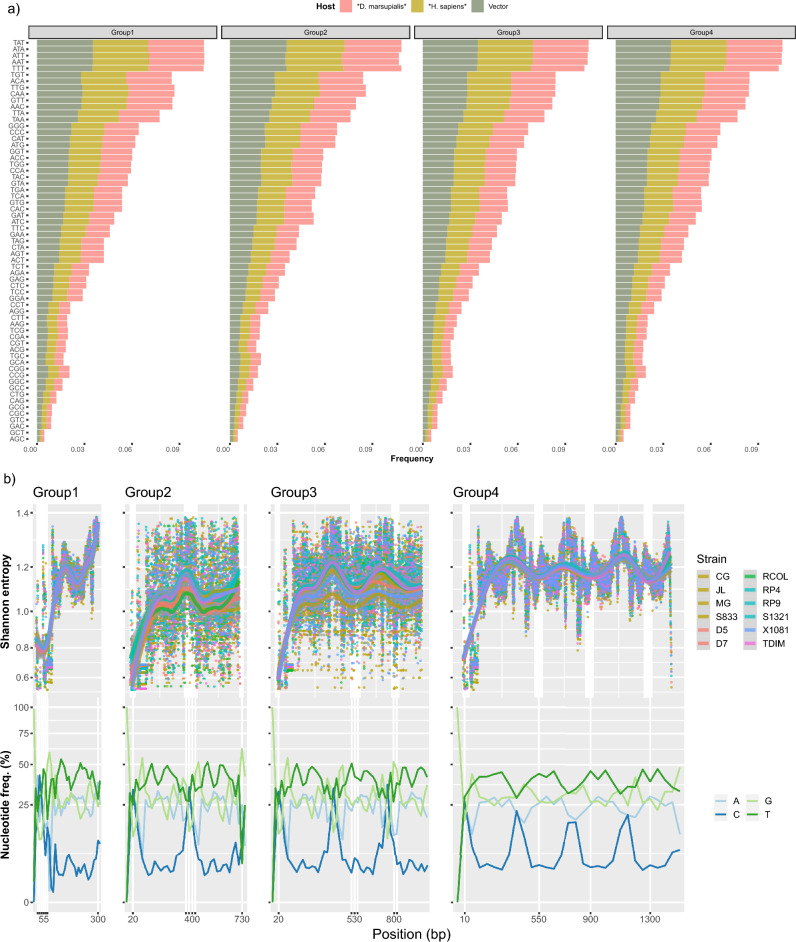
Minicirculome characterization of the (**a**) frequency of trinucleotide pattern, (**b**) Shannon’s entropy index and nucleotide composition frequency along minicircle sequences and groups identified in TcI clones of distinct strains as depicted in the inset.

The distribution of nucleotide composition exhibited a high degree of heterogeneity across the sequences, displaying variations both within and among groups. Additionally, we found a statistically significant difference in average of observed frequency nucleotide triplet pattern across minicircle groups (one-way ANOVA; *F* = 8.505, *P* < 0.001). Frequent nucleotide composition varied noticeably across multiple alignments of minicircle sequences (Fig. 2a,b and Supplementary Fig. S3). The most abundant tri-nucleotide patterns were AT-based, including ‘TAT,’ ‘ATA,’ ‘ATT,’ ‘AAT,’ and ‘TTT.’ These patterns were highly represented within the alignments of minicircle groups (Fig. 2a and Supplementary Fig. S3). Moreover, these patterns exhibited bias across all groups, with variations in the number of regions showing high abundance (Fig. 2b). Group 1 displayed a single, clearly defined DT-rich nucleotide region, while group 2 showed two such regions. In contrast, groups 3 and 4 featured three and four of these regions, respectively (Fig. 2b). The sequence diversity of minicircles revealed the presence of ‘moderately conserved’ regions, as defined by the Shannon's Entropy index, across different minicircle groups (Fig. 2b). The count of these regions ranged from one in the shortest minicircles (ranging from 331 to 400 bp in group 1) to two in group 2 (ranging from 690 to 760 bp), three in group 3 (ranging from 1.0 to 1.2 kb), and between four and five in group 4 (ranging from 1.4 to 1.6 kb) (Fig. 2b).

In summary, our comprehensive analysis of TcI mcDNA, considering minicircle length, abundance, nucleotide composition (specifically, DT-based trinucleotide distribution), and the number of mHCRs, has revealed a categorization into four distinct subpopulations of minicircles. Group 1, the second most abundant, comprises smaller minicircles (~ 350 bp) with one conserved and one DT-rich region. Group 2, the least frequent, features minicircles of approximately 700 bp with two conserved and two DT-rich regions. Group 3, less common than Groups 1 and 4, consists of approximately 1000 bp minicircles with three conserved and three DT-rich regions. Finally, Group 4, the most abundant, represents canonical minicircles with four moderately conserved regions, not strictly defined mHCRs, and four DT-rich regions, as illustrated in Fig. 2b and detailed in Table 2. This categorization offers valuable insights into the diversity and structural characteristics of minicircles within TcI mcDNA.

### Minicirculome ancestry in TcI clones

**Figure 3:**
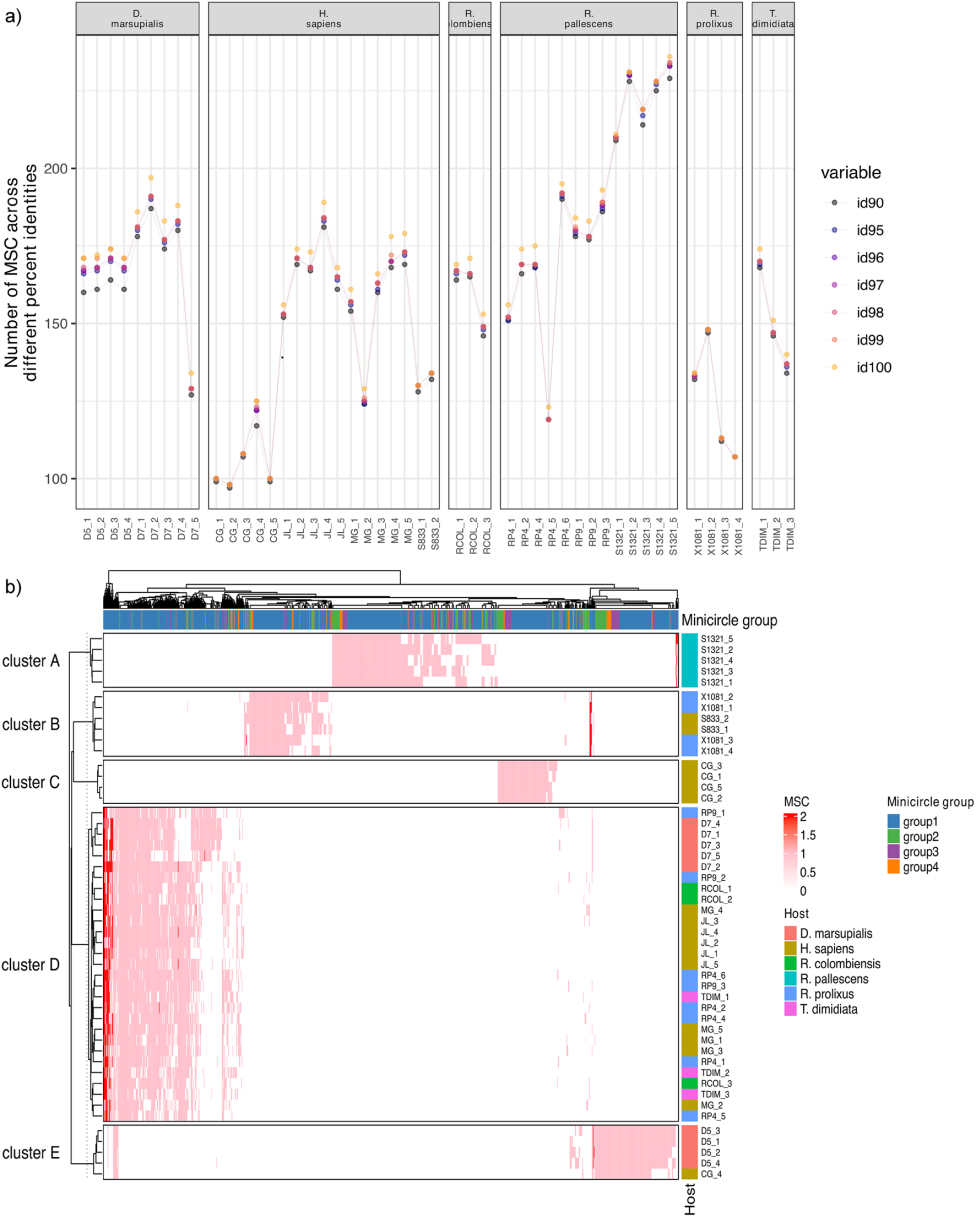
Number of minicircle sequence classes identified in TcI (**a**) Number of minicircle sequence classes (MSCs) identified at different percent identity in TcI clones from distinct strains and host species. (**b**) Minicircle sequence classes (MSCs) clusters identified in TcI isolated from distinct human, opossum, and vector species as denoted in the inset.

Taking into consideration the noticeable disparity in minicircle count observed in clone RP4_3 compared to all other isolates (Fig. 1), and after excluding potential artificial dimers, we conducted an investigation into minicircle sequence diversity using 8210 circular minicircles originating from 49 *T. cruzi* TcI clones. For clustering analyses, circular minicircles from all four groups were combined, resulting in the identification of a total of 1497 MSCs (minicircle sequence clusters) at 100% identity. This count decreased to 1063 MSCs at 99% identity and further reduced to 915 MSCs at 90% identity (Fig. 3a).

During the clustering process, the proportion of perfectly aligned minicircle sequences, characterized by alignments without any insertions or deletions, decreased progressively from 100% at 100% identity to 88% at 99% identity and to 74% at 90% identity (Supplementary Fig. S4). A significant majority of the identified MSCs were unique to a specific minicircle group (Supplementary Fig. S5). For example, at 99% identity, 11.19% of the MSCs were unique to group 2, 9.31% were unique to group 3, 5.17% were unique to group 4, and 67.83% were unique to group 1. The proportion of MSCs containing different groups of minicircles decreased from 11.04% at 90% identity to 6.49% at 99% identity and further declined to 4.41% at 100% identity (Supplementary Fig. S5).

Upon further examination of minicircle sequence diversity at 99% identity, it was observed that strain CG exhibited the lowest number of MSCs, with a mean of 106.2, while strain S1321 had the highest number of MSCs, with a mean of 224.4 (Fig. 3a). Analyzing the similarity of minicircle sequences, specifically the presence or absence of MSCs per isolate, allowed us to categorize all clones into five distinct clusters denoted as A, B, C, D, and E. Each cluster exhibited unique sets of MSCs (Fig. 3b). Cluster A (represented by S1321) contained 29.26% of its MSCs that were unique to this strain. In contrast, clusters C (CG) and E (D5) had 10.35% and 16.56% unique MSCs, respectively (Fig. 3b and Supplementary Fig. S6). It is worth noting that clone CG_4 displayed minicircle diversity similar to that of strain D5 (Fig. 3b and Supplementary Fig. S6).

Cluster B consisted of strains X1081 and S833, sharing the same set of MSCs, with 15.90% of the MSCs being unique to these isolates (Fig. 3b and Supplementary Fig. S6). Cluster D encompassed strains D7, JL, MG, RCOL, RP4, RP9, S1321, and TDIM, all sharing the same set of MSCs and featuring 26.06% unique MSCs (Supplementary Fig. S6). There was minimal overlap in minicircle sequence diversity between these clusters, with only 1.41% of the MSCs shared between clusters D and E, 0.38% between clusters B and D, and 0.09% between clusters B and E (Fig. 3b and Supplementary Fig. S6). Principal Component Analysis conducted on the same data confirmed these observations (Supplementary Fig. S7). Notably, the second axis of the PCA plot effectively differentiated all clusters, with the exception of clone CG_4, which closely grouped with D5.

### Minicirculome gRNA repertoire in *T. cruzi* clones

**Figure 4:**
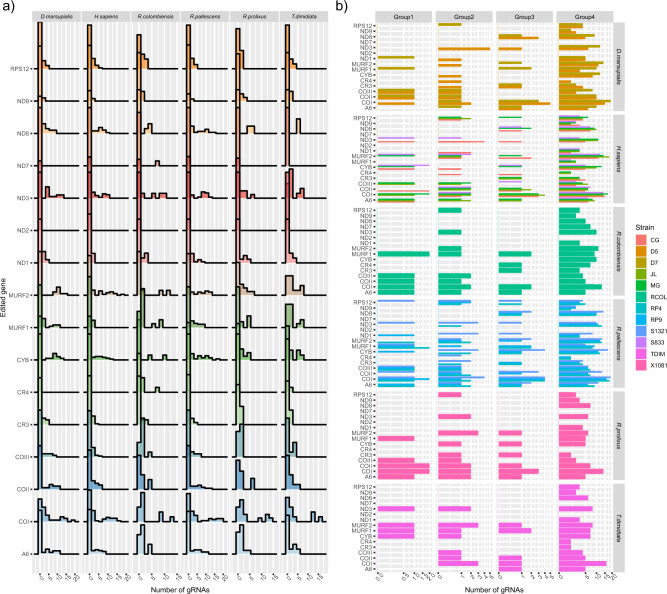
(**a**) *Trypanosoma cruzi* I minicirculome gRNA repertoire, and its respective edited gene are present in TcI strains from humans, *D. marsupialis*, and vectors. (**b**) As shown in the inset, several predicted gRNAs in the minicircle groups identified, and its respective edited gene in TcI strains from human, opossum, and vector hosts of distinct strains.

**Figure 5:**
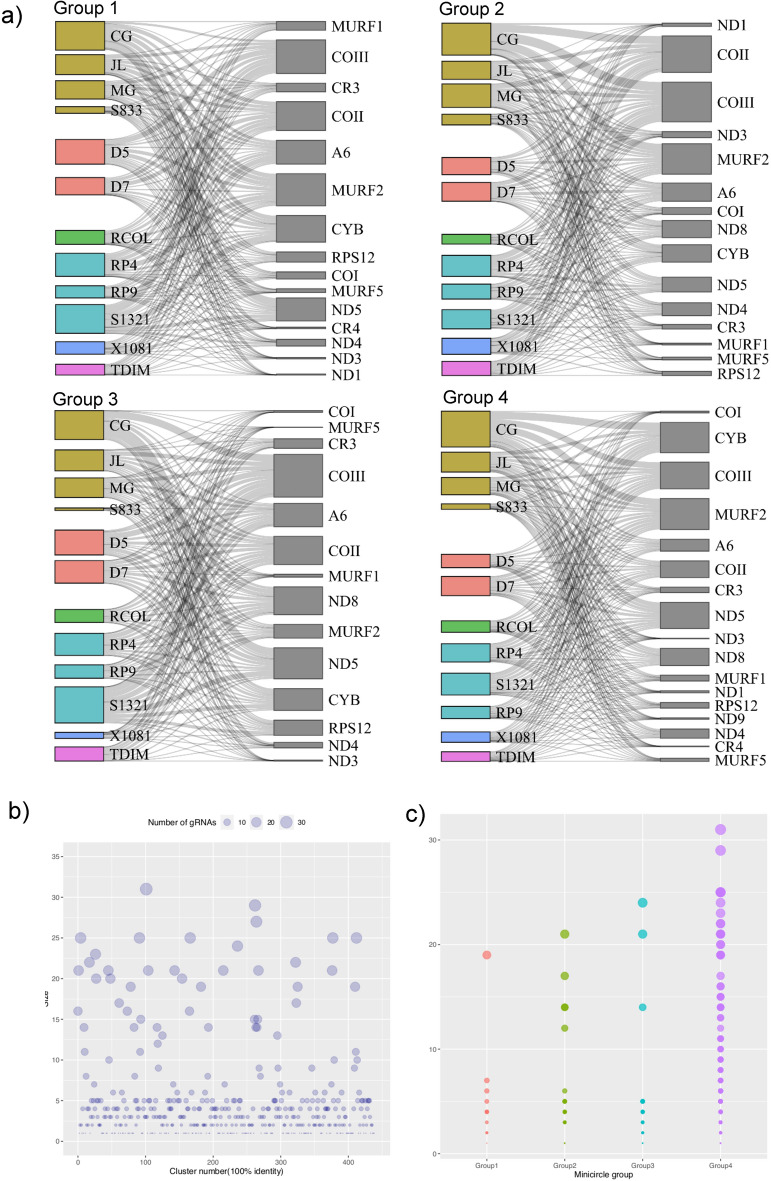
(**a**) The inset shows that the Sankey diagram for predicted gRNAs in minicircle groups unveiled in TcI and its respective edited gene in strains isolated from human, opossum, and vector hosts. (Web-based widgets are supplied as supplementary). Number of gRNAs clusters at 100% identity in the total minicirculome (**b**) and (**c**) in each minicircle group unveiled in TcI *clones.*

A total of 9849 guide RNAs (gRNAs) were predicted for 16 out of 18 tested genes, with no gRNAs found for the ND2 gene in any of the analyzed clones, whereas for the ND7 gene only one gRNA was found in a *R. colombiensis* clone (Fig. 4). Predicted gRNAs were identified for all genes across different host and strain origins (Fig. 4a). However, distinct differences in the number of predicted gRNAs for several genes were observed among clones from different strains (Fig. 4b). Excluding clone RP4_3 from *R. pallescens* RP4 strain, the proportion of the number of gRNAs was associated with the number of clones analyzed within each strain. For instance, the lowest proportion of gRNAs (313 gRNAs) was identified in the human S833 strain, which included two clones. In contrast, the highest number of gRNAs (1928 gRNAs) was detected in the CG strain, followed by *R. pallescens* S1321 (1312 gRNAs), where five clones were analyzed (Fig. 5a). Excluding clone RP4_3, the overall number of gRNAs per clone was 200, ranging from 110 in CG_2 to 272 in S1321_4 (Supplementary Fig. S8). No significant differences in the number of gRNAs were identified among strains (based on Kruskal–Wallis’ test, *P* value = 0.36, H statistics = 107) or hosts (*P* value = 0.49, H statistics = 102). However, it is worth noting that 51 out of 153 pairwise comparisons between genes were significant after post-hoc Dunn's test (*P* value < 0.05, following the Benjamini–Hochberg adjusted method) (see Table S1). In general, the number of gRNAs predicted for edited genes across all clones was higher for cytochrome oxidase subunit genes (COI, COII, and COIII), cytochrome B gene (CYB), and the unidentified reading frame 2 (MURF2), while it was lower for ND1 and ND3 in almost all strains (Fig. 4b).

When considering gRNA diversity along minicircle groups, notable differences were observed in the number of predicted gRNAs and the abundance of edited genes among strains (Fig. 5a). For the most abundant minicircle group, Group 4, a total of 8562 gRNAs were predicted for all edited genes (with the exception of ND2), while Group 1 had 802 gRNAs, Group 2 had 666, and Group 3 had 1135. It is important to note that, aside from the ND2 gene, predicted gRNAs for all genes were present in Group 4 (Fig. 5a,b).

Moreover, for Group 1, no predicted gRNAs were found for the ND8 and ND9 genes, and the same was true for the ND9 and CR4 genes in Group 2, as well as the ND1, ND9, and CR4 genes in Group 3 (Fig. 5a,b). Similarly, the number of predicted gRNAs for editing genes varied among minicircle groups as well as among clones from different strains and hosts (Supplementary Fig. S8). Null or low numbers of gRNAs (fewer than 3) were predicted for editing various genes in several clones (Fig. 4b).

**Figure 6:**
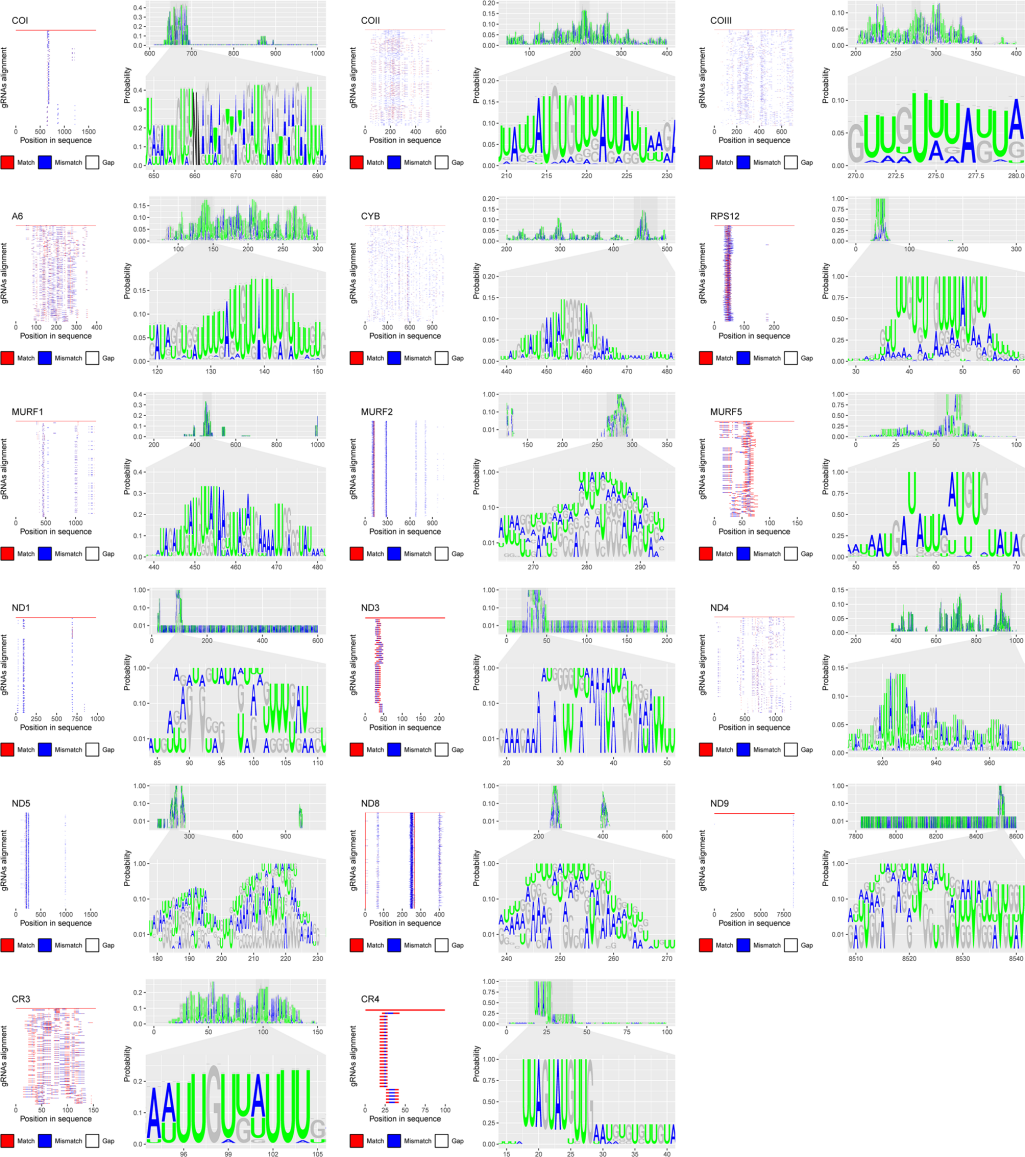
Mitochondrial mRNA editing positions inferred from the gRNA repertoire in TcI strains from humans*, D. marsupialis*, and vectors. The left panel depicts a schematic alignment of total gRNAs against their respective gene-consensus, while the right panel illustrates specific positions of editing as a web logo. In the web logo, only AU nucleotides are colored, and ambiguous ‘N’ residues are masked as transparent.

The functional gRNA repertoire reveals a wide diversity in the gRNA editing process along the sequences of all genes (Fig. 6). Notably, well-conserved editing positions were identified in several genes, including COI, RPS12, CR3, MURF1-2, and all NADH dehydrogenase subunits (except ND4). In contrast, diverse editing regions (called “cascades” elsewhere^15^) were observed for ATPase (A6), COII, COII, CYB, and CR3 (Fig. 6). Throughout the gRNA alignments, regions with moderate to high frequencies of AU were consistently observed. However, the presence of regions containing CG at high frequencies may be contingent on clone/gene-specific editing.

A total of 439 clusters of gRNAs at 100% identity were identified in the entire dataset, with 32.3% (142) consisting of single sequences. The mean cluster size was 4.5 sequences, and 22 clusters contained over 20 and 31 sequences (Fig. 5b). Concerning minicircle groups, the number of clusters was notably higher in Group 4, where 378 clusters were identified (Fig. 5c).

The most abundant cluster, referred to as gRNAs90, was exclusively found in Group 4, encompassing 31 clones from nine strains originating from distinct hosts. The centroid sequence of gRNAs90, “ATTGTGACTTAGATAGTTCAAGTTGTTAGTTAGAT,” is involved in COII mRNA gene editing. Additionally, among the 439 clusters identified, six were shared between groups, involved in editing several positions of genes such as MURF2, COIII, ND5, and CYB (Supplementary Fig. S9). Notably, for the CYB gene, two predicted gRNAs, editing distinct gene regions, were found in both Groups 3 and 4 simultaneously (Supplementary Fig. S9).

## Discussion

The analysis of maxicircles revealed low diversity among most clones and strains, providing moderate phylogenetic resolution within TcI at the clonal-strain level (Fig. 1). The phylogenetic tree indicated that clones from four out of 12 strains (X1081, S132, D5, and CG, except for clone CG_4, which clustered within the D5 clade) formed a well-supported clade. While the entire tree did not show a conclusive association with the biological origin (whether from humans, mammalian hosts, or vectors), monophyletic clades containing sylvatic strains X1081, S132, and D5 were evident. This finding aligns with a previous phylogeny based on the entire nuclear genome. Furthermore, these concordant results suggested that clone CG_4 is clustered into the D5 clade, in accordance with evidence of multiclonality in natural populations. This correspondence in phylogenetic signal between the nuclear and maxicircle genomes has also been reported in several strains of *T. cruzi* from distinct Discrete Typing Units (DTUs), indicating similar trends and inheritance patterns occurring in both nuclear and maxicircle genomes. This parallelism with *T. brucei*, where kinetoplast DNA (kDNA) maxicircles exhibit uniparental inheritance with no detectable evidence of heteroplasmy, underscores the significance of these findings^22^.

The number of minicircles per clone was found to be consistent among clones and was not influenced by sequencing depth or the number of high-quality mapped reads. Specifically, the total number of minicircles, often referred to as circularized minicircles, that constitute the mitochondrial DNA (mcDNA) per clone averaged 165, ranging from 124 to 204 minicircles per clone, and no significant differences were observed among strains or based on the biological origin of the clones (Figs. 1b, 2)^10^. While previous studies did not provide evidence regarding the number or abundance of minicircles per clone, a recent report indicated that the Y strain (TcII) possesses at least 286 minicircles in its mcDNA^10^. It is important to note that the overall number reported here is approximately 42% lower than what was reported for the Y strain. However, this difference could be attributed to the potential multi-clone inheritance of the mcDNA in the Y strain and evolutionary divergence between TcI and TcII lineages^10^.

Minicircle abundance analysis indicated that, on average, about 9.6% of copies per haploid genome are present in each cell, with 45.6% existing as single copies. Most of the remaining minicircles are likely present in quantities ranging from 2 to 50 copies. Interestingly, a few highly-abundant minicircles (exceeding 1500 copies) were identified in some clones isolated from humans and vectors, suggesting that certain minicircles are overrepresented^10^. The process of mcDNA replication and the potential effects of factors such as density (abundance), diversity, and other intra-genomic elements like drift, mutation rates, and even potential intra-individual functional selection are still unknown. Some theoretical models propose that the probability of forming a highly linked network increases linearly with minicircle density^23^, and the existence of “silent” mHVRs (non-coding gRNA hypervariable regions) reported in several trypanosomes supports the idea of minicircle drift as a consequence of random segregation^22,24,25^. Unfortunately, our results do not provide conclusive evidence to support any specific hypotheses regarding the causes of abundance variation or the origins of mcDNA. Further analyses are needed to elucidate the intricate relationship between diversity, abundance, and function within the complex minicircle network.

In summary, we have identified four distinct subpopulations of minicircles within the mitochondrial DNA (mcDNA) of TcI. The existence of these minicircle "subpopulations" was initially suggested back in 1984 by Frasch et al.^26^ and Sanchez et al.^27^, and more recently, confirmed by Callejas-Hernández et al.^10^ based on deep sequencing. The latter study, focused on the Y and Bug2148 strains (TcII and TcV, respectively), concurred with our findings regarding the length and number of “mHCRs” in these minicircles^10^. However, our study goes further by providing a more detailed description of minicircle subpopulations at both clonal and population levels. Interestingly, we found that the “mHCRs,” while not ultra-conserved, are more conserved than other regions on minicircles. Additionally, our analysis of AT trinucleotide composition revealed variations in the number of mHVRs among these subpopulations, which in turn could result in differences in the number of regions encoding guide RNAs (gRNAs). This observation supports the hypothesis of the existence of “silent” mHVRs, as suggested elsewhere^10,24,25^. Previous research on mHVRs has focused on diversity, inheritance, and gRNA repertoire using amplicon deep sequencing of mHVRs^10^. These studies have revealed variable numbers of mHVR clusters across different *T. cruzi* lineages, with some clusters shared among strains within and between lineages. However, our results suggest that the number of reported mHVR clusters alone may not fully represent the minicircle count or abundance within a cell. Instead, they provide insights into potential functional classes of minicircles encoding gRNAs. Further research is needed to elucidate the functional roles of these minicircle subpopulations in *T. cruzi*.

Regarding the minicircle sequence classes (MCSs) found in the canonical subpopulation of mitochondrial DNA (mcDNA) across all clones analyzed, our results do not reveal conclusive differentiation among MCSs in the majority of clones, whether within the same strain or across different strains (Fig. 3a). The high degree of mixed MCSs observed in minicircles is consistent across clones from distinct hosts, diverse ecosystems, and distant geographical regions. This pattern likely reflects the ancient enzootic cycle of *T. cruzi* I in Colombia. Furthermore, the presence of shared MCSs in some clones, whether from the same or different strains and varied origins, suggests the possibility of random segregation. This implies that minicircles do not have a single, clear-cut heritage origin. Previous suggestions of bi-parental inheritance of minicircles in *T. cruzi* have been bolstered by more recent confirmation in experimental hybrids of *T. brucei*^5,19^. In light of these findings, we conclude that, in contrast to observations in nuclear DNA and maxicircles, the ancestry of mcDNA reveals multiple hybridization events occurring in TcI across diverse host-infecting populations. It is important to note that the clones we analyzed were isolated from strains collected many years ago (between 13 and 25 years ago). Therefore, additional research is needed to further support the notions of random segregation and bi-parental inheritance in mcDNA, particularly in clones from more recently isolated strains.

Our analysis of the gRNA repertoire within mcDNA has revealed significant differences in the presence of distinct gRNAs among clones originating from various strains and host species. Interestingly, we have identified identical gRNA clusters in different clones, and we observed six gRNAs shared between minicircle groups. In contrast to variations in the number of gRNAs among clones, the genes that were predicted to have the most gRNAs were generally consistent across both clonal and minicircle subpopulations (Fig. 4). This suggests a degree of stability in gRNA presence among these genes. Our investigation has also unveiled evidence for potential trans-splicing processing in all maxicircle genes, with the exception of ND2. Furthermore, we propose that this process is not evenly balanced for all genes, as a significant portion of the predicted gRNAs was associated with cytochrome oxidase genes and MURF2. This observation leads us to speculate that the abundance of gRNAs for these genes may be linked to their high levels of transcriptional activity, as has been previously reported^15^.

Furthermore, here we support the prominent functional role for the canonical minicircle (i.e., group 4) at a cell scale but also suggest other minicircle groups could play other structural roles, such as kDNA structure or stability. Further studies about the specific functional or structural role of the no-canonical minicirculome are still needed. The number of gRNAs in the entire minicirculome was quite half of the minicircles found, which supports the presence of “silent” mHVRs^15,24,25^. Our results indicate that distinct gRNAs are coded for the same mRNA in a single clone. No specific gRNAs are found in certain subpopulations, nor both strains or origins. These results support the hypothesis that multiple gRNAs can drive trans-splicing cascades for some mitochondrial genes^15^.

## Materials and methods

### *Trypanosoma cruzi* I cloning, sequencing, and WGS genome mapping

We obtained a total of 48 clones derived from 12 different strains, which were isolated from various sources, including humans, sylvatic hosts, and triatomine insects from both Colombia and Brazil (Table 1). It is worth noting that among these clones, 15 were isolated from four strains that had previously been analyzed for genome structure by Cruz-Saavedra et al.^22^. Our sequencing workflow involved cell cloning and sorting, followed by DNA extraction, library preparation, and Illumina HiSeq X-Ten sequencing as previously described by Cruz-Saavedra et al.^22^. We subjected the paired reads to a base correction for mismatched base pair (*q* < 15), and removed any polyX tail using fastp v.0.20.1^28^. These reads were then aligned to the *T. cruzi*—Brazil A4v49 genome reference^29^ using BWA-mem v0.7.3 with default parameters^30^. Any reads that remained unmapped were preserved for subsequent identification of kDNA maxicirces and minicircles.

### Maxicircle assembly and phylogeny of Colombian TcI clones

We assembled the coding regions of maxicircles using MEGAHIT^31^ with default parameters for a kmer range of 29, 49, 69, 89, 109, and 119, as recommended (https://frebio.github.io/komics/). To identify the maxicircle coding regions for each clone, we performed BLAST searches against the complete maxicircle sequence of *T. cruzi* I—Strain Dm28c (MW421590)^9^. The retained sequences for all clones were aligned using the “auto” function in MAFFT v7.205^32^. Subsequently, the coding regions were used to construct Maximum Likelihood (ML) trees on a per-gene basis. This was done using RAxML^33^, with the GTRGAMMA substitution model, which was determined as the best-fitted model after analyzing each gene dataset separately with ModelGenerator v0.85^34^. We generated one hundred bootstrap pseudoreplicates, which were then mapped onto the most likely tree topology.

### Assembly and ancestry of minicircles

Minicircle analysis was conducted using the Kinetoplast Genomics (KOMICS) automated Python package, a proven tool used for mitochondrial genome characterization in *Leishmania*^3^ and *Trypanosoma brucei*^6^. The KOMICS pipeline involves three main steps: assembly, circularization, and polishing. The assembly utilized MEGAHIT^31^ and extracted minicircle contigs based on the presence of the CSB-3 motif. Circularization employed BLAST^35^ to remove overlapping sequences at the start and end of minicircle contigs. Polishing realigned the contigs using the CSB3 motif and ensured alignment by placing the CSB1 motif at the start. This modified version of KOMICS, designed to accommodate the multiple conserved regions in *T. cruzi* minicircles, is accessible online.

The KOMICS assemble and circularize commands were executed independently for each *T. cruzi* clone, with long kmer values (kmin 99 and kmax 129) employed during assembly. Assembly quality was assessed by examining the number of mapped reads to the extended minicircles in polished minicircles using BWA-mem^30^. Once circular minicircles were obtained, they were categorized into four groups based on their length^10^. The KOMICS polish command was run for each clone and minicircle group independently. Polished minicircles from all clones were combined for each group, and preliminary clustering analysis at 99% identity was performed using VSEARCH. To address partial overlaps in some minicircles during clustering, a custom Python script was employed to realign non-overlapping regions.

Subsequently, all circular minicircles from the four groups were combined, and VSEARCH was used at various percent identity thresholds (90, 95, 96, 97, 98, 99, 100) to explore the diversity of minicircle sequence classes (MSCs) across all clones. The rKOMICS package^36^ was employed to examine the number, similarity, and ancestry of MSCs for each clone, isolate, and host, providing a comprehensive analysis of minicircle sequence diversity and organization.

### Minicirculome characterization and structure

To accessing for mcDNA structure multiple sequence alignment of minicircles belonging each group was retrieved separately as derived from KOMICS. Tri-nucleotide composition (192 triplet pattern) frequency was addressed for each minicircle group and host using the *compseq* tool implemented in EMBOSS v.6.5.7^37^. Significance was tested by one-way ANOVA using *stats* v.4.1.0 R package. Nucleotide composition frequency across all bases in minicircle groups was calculated using seqPattern v.1.0^38^, and depicted in *fastqcr* v.0.1.3 package. Shannon entropy index was calculated to address for nucleotide variation in minicircles using the public-available entropy*.R* script (https://github.com/MiguelMSandin/DNA-alignment-entropy/).

### Minicirculome guide RNAs prediction and characterization

We predicted the gRNA repertoire for minicirculome in each clone based on the mRNAs of maxicircles genes by using a model profile using a Hidden Markov Model using HHMER v.3.3.2. (http://hmmer.org/) and local c++ script. Briefly, predicted edited mRNA per clone was obtained by Smith-Waterman local alignment of maxicircle’ DNA sequences against the *T. cruzi* I—Strains Silvio (FJ203996) and SC43 (MT554701) annotated mRNA for 18 mitochondrial CDS as follows: unidentified reading frames 1, 2 and 5 (MURF1, MURF2 and MURF5), NADH dehydrogenase subunits 1, 3–5, and 7–9 (ND1, ND2, ND3, ND4, ND5, ND7, ND8 and ND9), C-rich regions 3 and 4 (CR3 and CR4), ATPase6 (A6), cytochrome b (CyB), ribosomal protein S12 (RPS12), cytochrome oxidase subunits 1, 2 and 3 (COI, COII and COIII) as reported elsewhere^9,15^. For each alignment, a majority consensus inserting all uridines (U's) at the sites in the mRNA was retrieved per each clone using EMBOSS v.6.6.0 toolkit (The European Molecular Biology Open Software Suite 2000). We built the HMMER profile for each gene, including all predicted edited mRNA of 50 clones using *hmmbuild* function in HHMER v.3.3.2. (http://hmmer.org/). Predicted gRNAs were assessed to each minicircle type (i.e., circularized minicircles groups) using *hmmaling*. The alignment per gene/clone was processed by masking gapped and low posterior probability positions (< 0.9) using *esl-alimask* (http://hmmer.org/)*.* Sequences with a length less than 1% fraction of the length of the median length sequence in the alignment and with a length less than 10% of the aligned residues were removed using *esl-alimanip* (http://hmmer.org/). In all cases gRNAs lower to 10 bp were removed. Predicted gRNAs-mRNA alignments were conducted in order to identified editing cascades using BWA-mem v0.7.3^30^. Resulted gRNAs for total minicirculome was clustered using 100% identity and cluster centroids sequence were retrieved using VSEARCH^39^. Finally, to assess the functional traits of the gRNAs repertoire the complete dataset of gRNAs was aligned against its respective gene's consensus (i.e., majority consensus) using *seqvisr* v.0.2.7, and nucleotide editing logo was generated using the *ggseqlogo*^40^ R packages.

### Supplementary Information

Supplementary Information 1.

Supplementary Information 2.

## Data Availability

Data generated in this work were deposited on ENA under the bioproject PRJEB48841. All supporting data were included in supplementary data files, eleven supplementary files are available with the online version of this article.
