# Supplementary material for: High-throughput analysis of the Trypanosoma cruzi minicirculome (mcDNA) unveils structural variation and functional diversity
Source: Sci Rep. 2024 Mar 7;14:5578. doi: 10.1038/s41598-024-56076-4 (PMC10917808; doi:10.1038/s41598-024-56076-4)

**Supplementary information**

**Trypanosoma cruzi I: High-throughput analysis of the minicirculome (mcDNA) unveils structural variation and functional diversity**

Andrés Gómez-Palacio^1^, Lissa Cruz-Saavedra^2^, Frederik Van den Broeck^3,4^, Manon Geerts^5,6^, Sebastián Pita^7^, Gustavo A. Vallejo^8^, Julio C. Carranza^8^, Juan David Ramírez^2,9^*


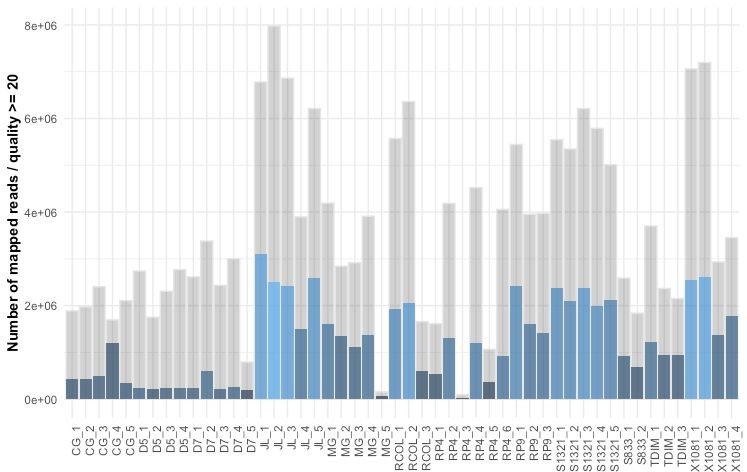
**Supplementary** **Fig. 1.** The number of mapped and high-quality mapped reads (Q >= 20) in minicircles contigs assembled (colors of the bars are depicted based on the number of mapped reads). The exact origin of clones is shown in Table. 1.

**Supplementary Fig. 2.** Minicirculome length distribution along 50 TcI clones.


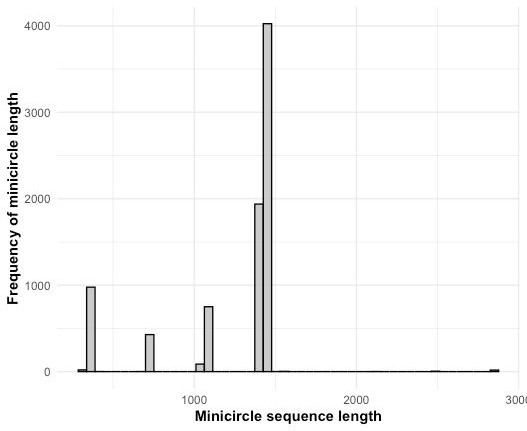


**Supplementary Fig. 3.** The density of highly frequent tri-nucleotide composition of minicircles sequences in groups 1 (blue), 2 (green), 3 (purple), and 4 (orange) defined according to TcI.


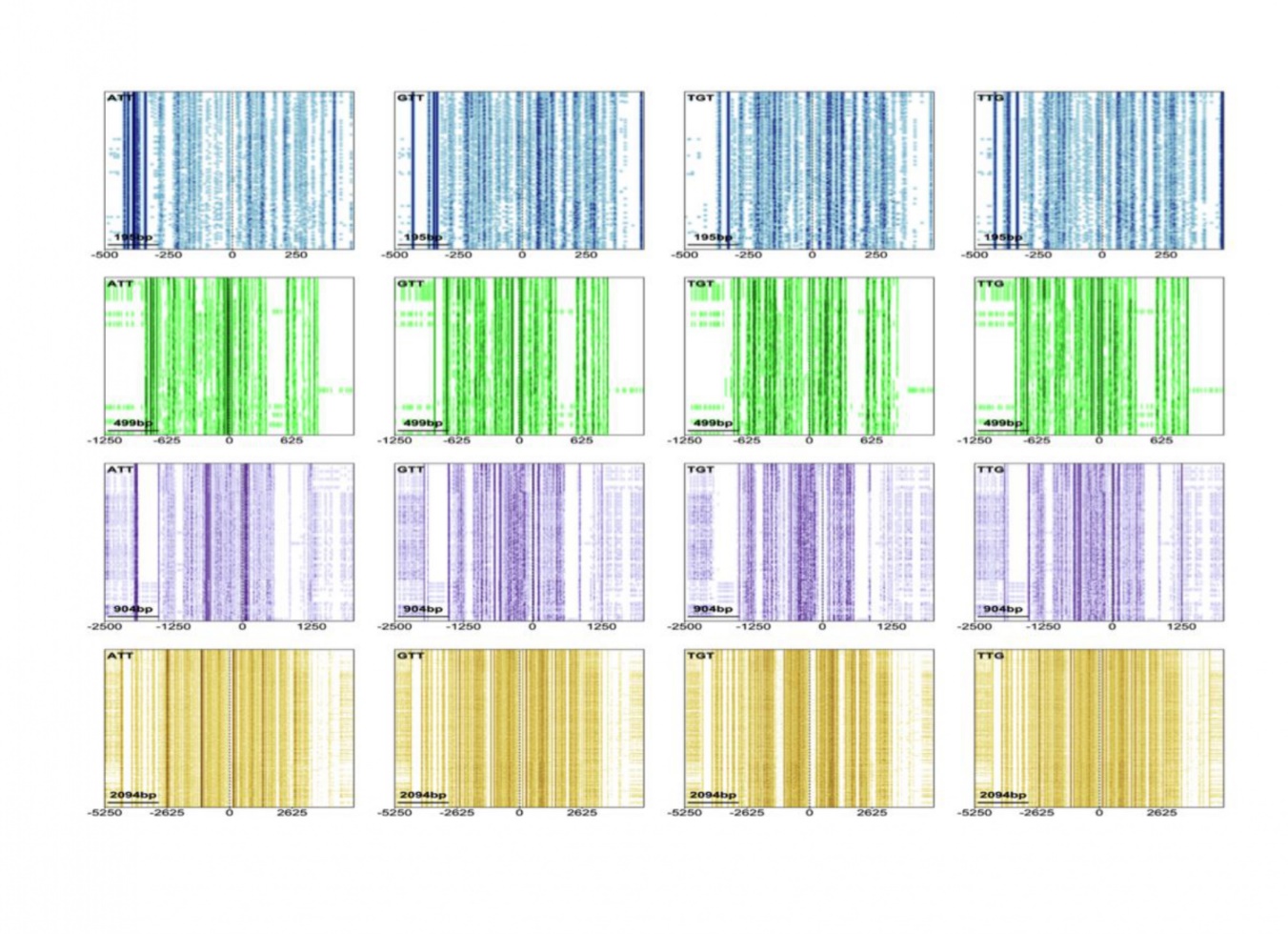


**Supplementary Fig. 4.** The number of MSCs (blue) and proportion of perfect alignments (red) were obtained following clustering analyses for a range of percent identities in TcI clones.


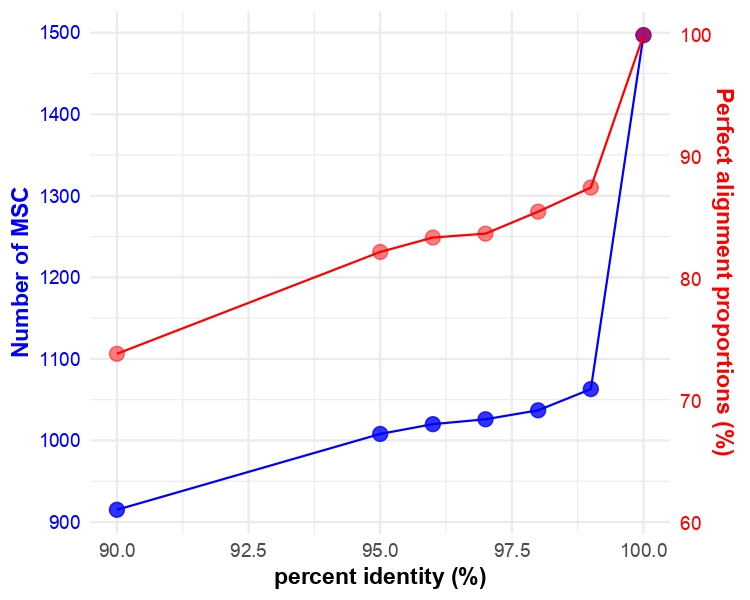


**Supplementary Fig. 5.** Relative abundance of unique and shared minicircle sequence classes (MSCs) identified at different percent identity in minicircle groups of TcI clones.


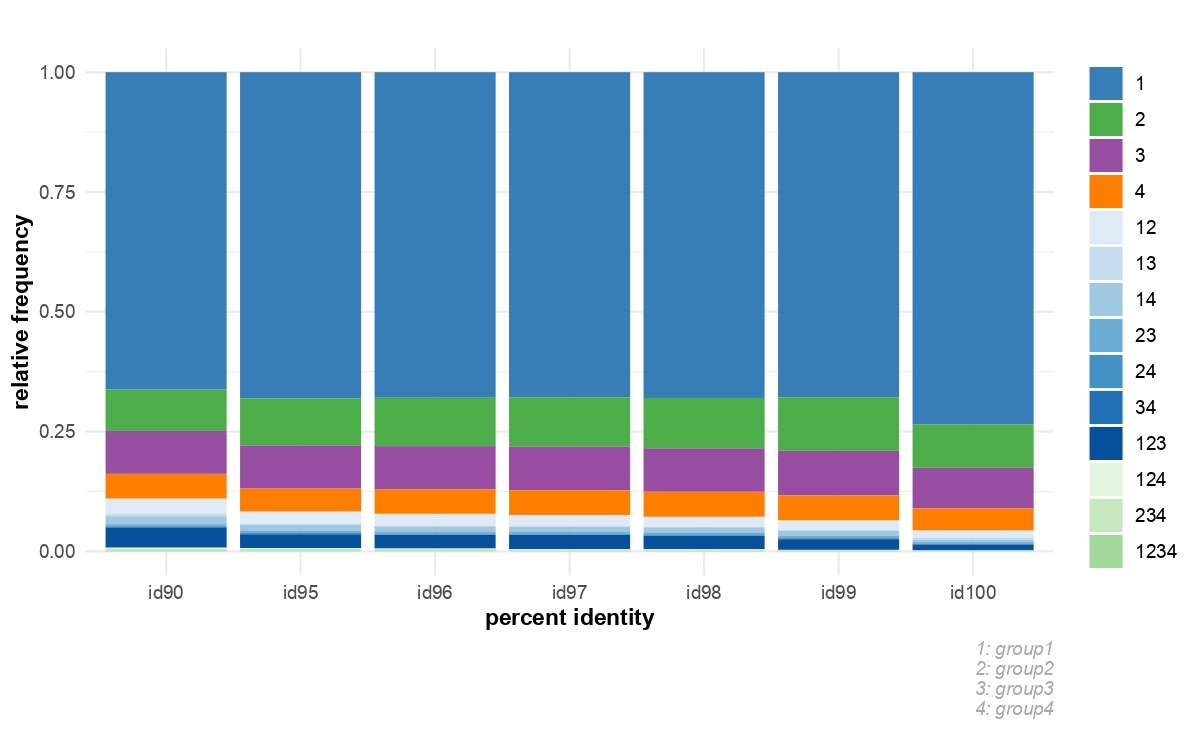


**Supplementary Fig. 6.** Relative abundance of Minicircle sequence classes (MSCs) clusters present identified at different percent identity across all TcI clones


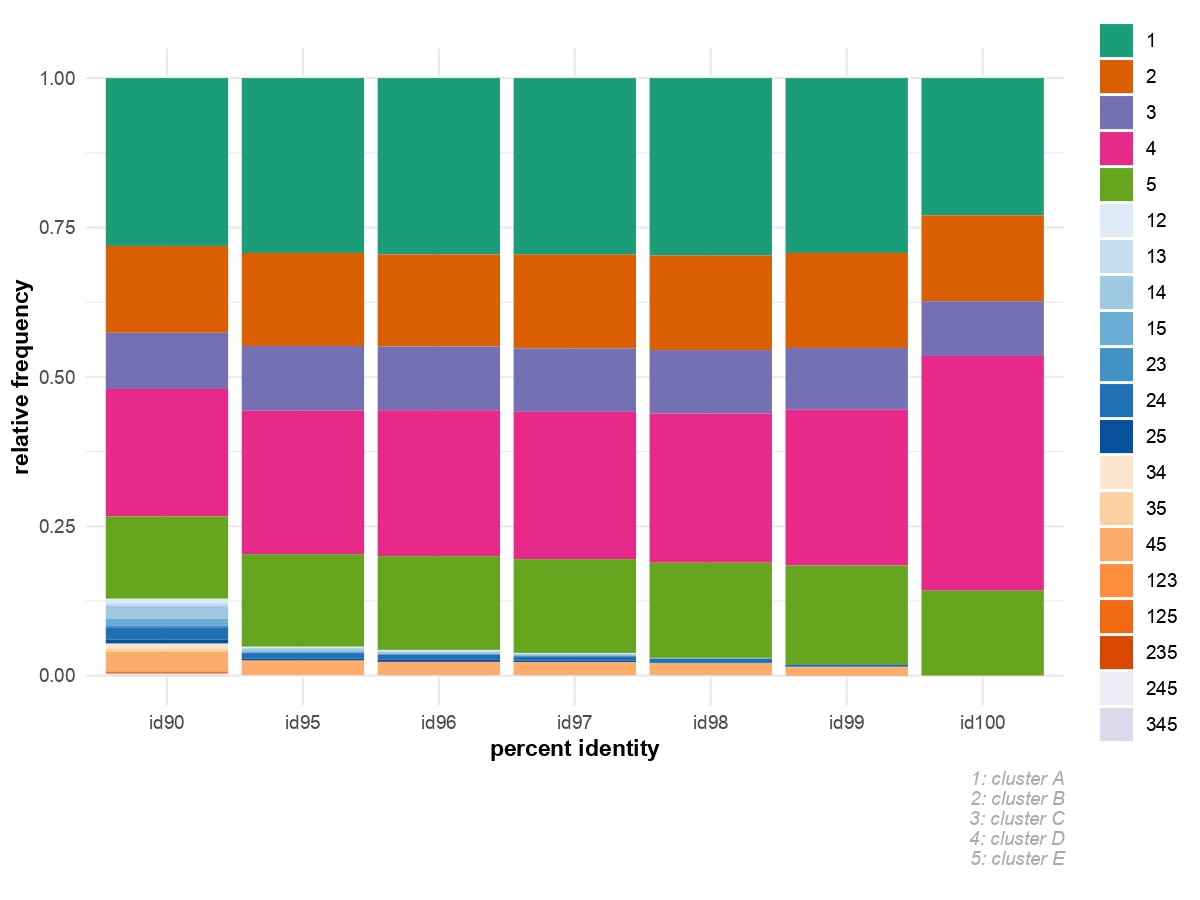


**Supplementary Fig. 7.** Principal component analysis (PCA) based on minicircle sequence classes (MSC) in TcI clones isolated from distinct human, opossum, and vector species as colored in the inset.


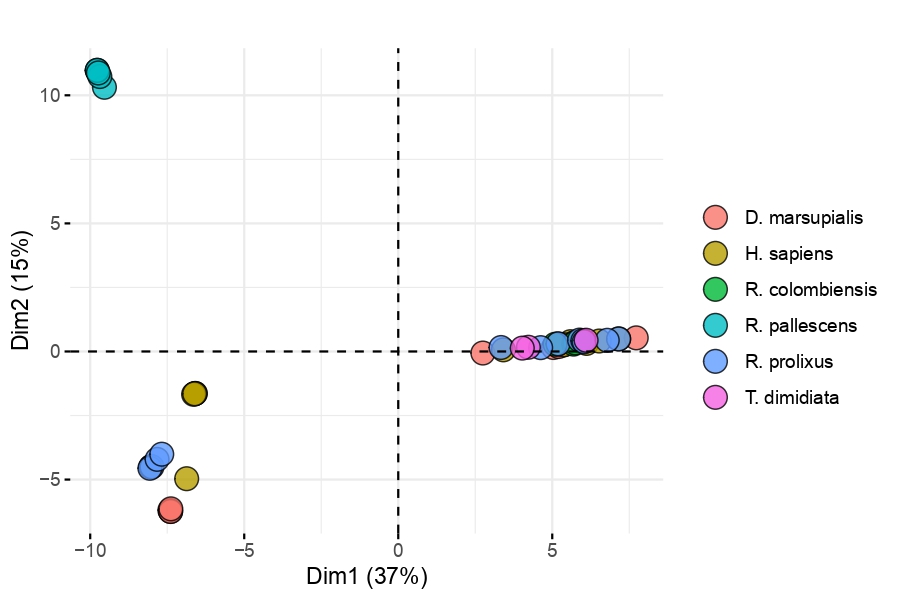


**Supplementary Fig. 8.** The total number of gRNA per clone in TcI clones from human, opossum, and vector species.


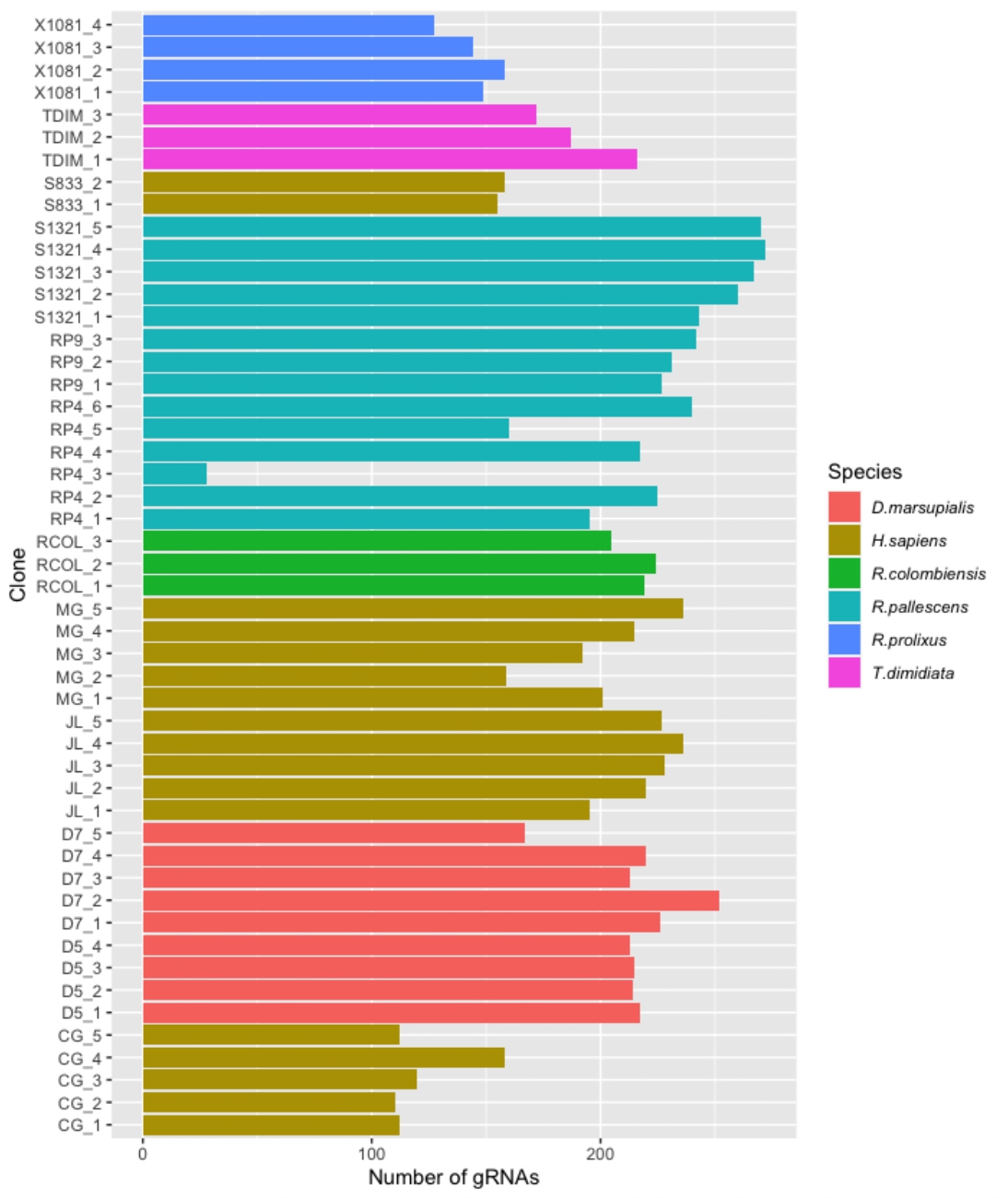


**Supplementary** **Fig. 9.** Nucleotide alignment for 6 identical predicted gRNAs shared between minicircles groups against their reference genes of T. cruzi I – SILVIO Strain.


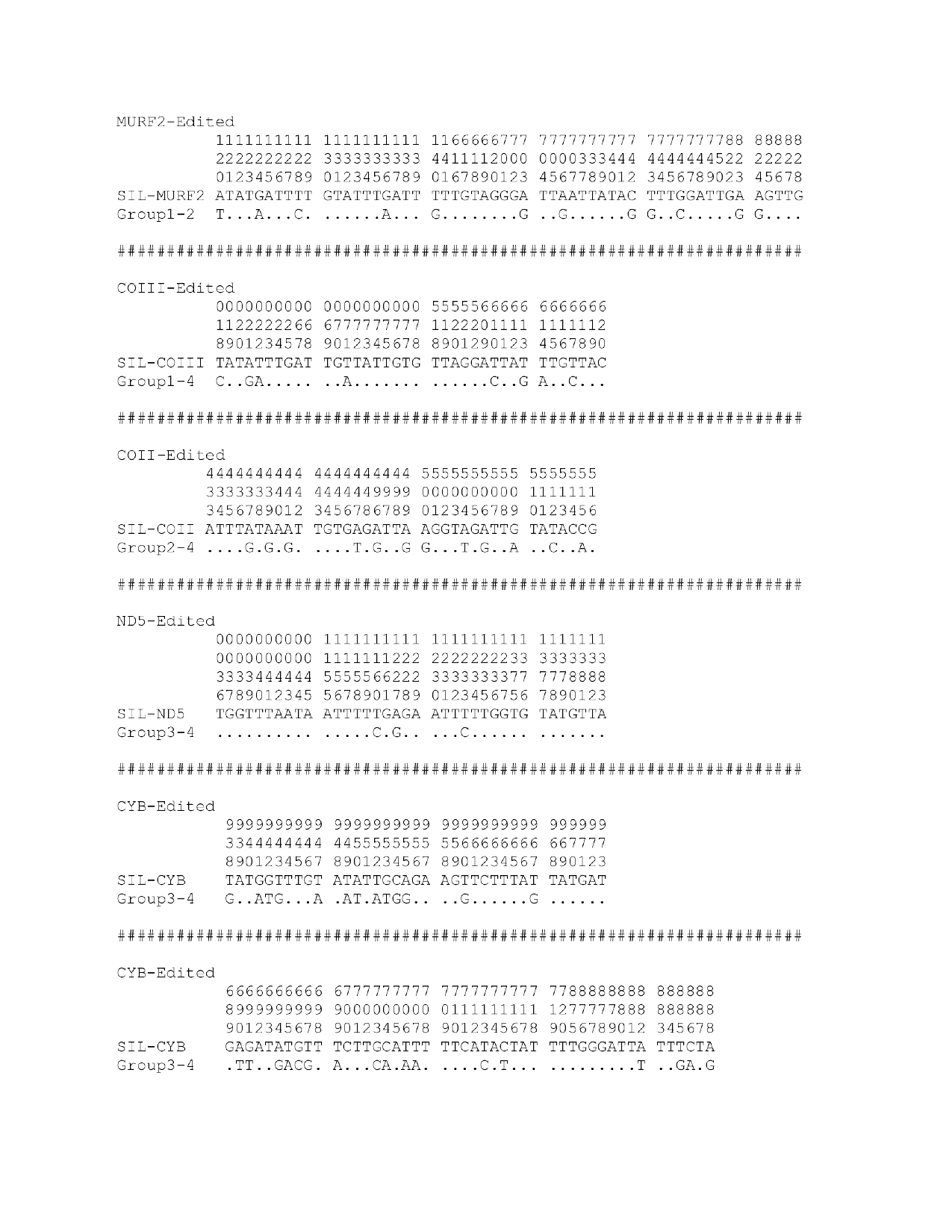

Supplement: Supplementary file 1 — Supplementary Information 1. [file 41598_2024_56076_MOESM1_ESM.docx]
